# Effects of S Doping and Oxygen Vacancy on the Physical Properties of Rutile TiO_2_ for Photocatalysis Applications Based on Density Functional Theory Study

**DOI:** 10.3390/ma18081688

**Published:** 2025-04-08

**Authors:** Fikadu Takele Geldasa, Francis Birhanu Dejene

**Affiliations:** Department of Chemical and Physical Sciences, Walter Sisulu University, Private Bag X1, Mthatha 5117, South Africa; fdejene@wsu.ac.za

**Keywords:** rutile TiO_2_, photocatalysis, absorption, bandgap, refractive index

## Abstract

This study explores the effects of sulfur (S) doping and oxygen vacancy (OV) creation on the fundamental properties of TiO_2_, which plays a crucial role in photocatalysis applications. Using density functional theory (DFT + U), we investigate how S doping and OV impact the structural, electronic, mechanical, and optical properties of rutile TiO_2_. The structural results reveal that the lattice constants of undoped rutile TiO_2_ are a = b = 4.63 Å and c = 2.98 Å, which are consistent with reported values. Upon S doping at concentrations of 6.25%, 12.5%, and 18.75%, the lattice constants expand to a = b = 4.89 Å, 5.14 Å, and 5.31 Å, and c = 3.27 Å, 3.69 Å, and 3.82 Å, respectively. This expansion is attributed to the difference in atomic radii between sulfur and oxygen atoms. In contrast, the presence of OV leads to a reduction in the lattice constants, with values of a = b = 4.17 Å and c = 2.82 Å. Our findings on the electronic properties indicate that both S doping and OV contribute to an improvement in the electronic structure, notably shifting the electronic bandgap toward the visible spectrum. Moreover, the mechanical properties show that S doping increases the material’s rigidity, while the introduction of OV results in a reduction of mechanical strength. This highlights a trade-off between improved photocatalytic activity and material durability. Lastly, the optical properties exhibit a red-shift in absorption due to S doping and the formation of OV, offering valuable insights for designing efficient photocatalysts for visible-light-driven applications.

## 1. Introduction

Titanium dioxide (TiO_2_) has a long history in the development of photocatalysis technology for applications such as water splitting and environmental remediation. It continues to play a significant role in various fields due to its characteristics, including chemical stability, non-toxicity, and favorable optical and electrical properties. TiO_2_ has important applications across a range of industries, including medicine, pollutant degradation, antibacterial treatments, self-cleaning, drug delivery, electrical energy storage, solar cells, optical coatings, gas sensors, and optoelectronic devices [1,2]. Despite its widespread use in photocatalysis, TiO_2_’s large bandgap limits its efficiency under visible light, restricting its practical applications in solar-driven processes such as pollutant degradation and water splitting [3,4,5]. As a result, significant efforts have been made to modify TiO_2_’s electronic structure to enhance its photocatalytic performance under visible light. One promising approach to tuning TiO_2_’s properties is doping with metallic or non-metallic elements, which has been shown to narrow the band gap and improve visible light absorption. The primary goal of doping is to modify the bandgap of the host material, either by decreasing or increasing its energy bandgap [4]. Additionally, the creation of oxygen vacancies in TiO_2_ can further alter the electronic structure, facilitating charge carrier separation and enhancing photocatalytic activity.

Rutile, anatase, and brookite are the three main polymorphs of TiO_2_, with rutile being the most stable at high temperatures [6,7]. Therefore, in the present work, the rutile phase is selected due to its stability compared to the other TiO_2_ phases. The rutile phase has a tetragonal structure with a bandgap greater than 3 eV, which limits its application in visible-light-driven photocatalysis [8]. Numerous theoretical and experimental studies have been conducted on the effects of metallic or non-metallic doping and oxygen vacancies on bandgap tuning and other physical properties of different TiO_2_ polymorphs for applications such as photocatalysis, pollutant degradation, solar cells, and sensors. Diao et al. [9] synthesized fluorine-doped rutile TiO_2_ nanorod arrays using a hydrothermal method for formaldehyde removal. Their study found that fluorine doping induced oxygen vacancies in rutile TiO_2_, providing more active sites for photocatalysis. Negi et al. [10] reported carbon-doped TiO_2_ nanoparticles synthesized via the sol-gel method for visible-light-driven photocatalytic activity. Their study showed that carbon doping significantly reduced the bandgap from 2.96 eV to 2.37 eV. Arunmetha et al. [11] prepared S-doped TiO_2_ nanoparticles from rutile sand and characterized their performance for hybrid solar cells. Their results indicated that S doping shifted the optical absorption from the UV to the visible region, enhancing photogenerated charge carriers. Mizukoshi et al. [12] investigated the visible light response of sulfur-doped rutile TiO_2_ photocatalysts produced by anodic oxidation for methylene blue degradation under visible light irradiation. Their study showed that sulfur doping played a significant role in improving the visible light response by narrowing the large bandgap of rutile TiO_2_.

Moreover, theoretical studies have also confirmed that visible-light-driven photocatalysis can be achieved through doping, which narrows the band gap of pristine TiO_2_. For instance, Chen et al. [13] utilized density functional theory (DFT) to investigate carbon and chromium co-doped rutile TiO_2_. Their study showed a larger redshift in the optical absorption edge for the C/Cr co-doped material compared to the single-doping materials, due to a significant reduction in the bandgap. Wang et al. [14] also employed DFT to investigate the electronic distribution of boron and yttrium co-doped TiO_2_. Their DFT calculations revealed that boron doping redistributed the O 2p electron states, while yttrium 5d states captured electrons, thus reducing charge carrier recombination. Gul et al. [15] examined the effects of the relative positions of Fe and N doping on the structural and other properties of TiO_2_ using a DFT approach. Their results indicated that Fe substitution at Ti sites and N substitution at O sites enhanced the photoelectrochemical properties of TiO_2_. Heffner et al. [16] reported on boron-doped and carbon-boron-co-doped TiO_2_(B) using DFT. This study found that impurity states were introduced after boron doping and carbon-boron co-doping, which improved the optical properties and made the material suitable for use as a photoelectrode. Thongyong et al. [17] combined experimental and density functional theory approaches to investigate the enhanced giant dielectric properties of Ni^2+^ and Ta^5+^ co-doped TiO_2_.

Similarly, oxygen vacancy (OV) can act as charge carrier centers and influence the photocatalytic properties by altering the electronic structure. Oxygen vacancy is the deficiency of oxygen in the crystal lattices of rutile TiO_2_ and it can enhance the photocatalytic activity of TiO_2_ by generating charge carriers active sites [18]. The effect of oxygen vacancy on the physico-chemical properties of TiO_2_ is studied by using both DFT and experimental approaches. One of the basic advantages of DFT for studying the oxygen vacancy is its simplicity in understanding its effect on materials’ adsorption properties. Linh et al. [19] performed DFT calculations to identify the influence of oxygen vacancies on the adsorption of TiO_2_. According to this study, the introduction of oxygen vacancy produces excess electrons. Hinuma et al. [20] also performed DFT calculations for the formation of oxygen vacancy and the subsequent molecular adsorption on the surface oxides. Panta et al. [21] also utilized the DFT approach to investigate the hydrogen molecules’ adsorption on the Zr doped and oxygen vacancy rutile TiO_2_ surface. Their result shows that the adsorption energies of Zr doped and Zr doped oxygen vacancy rutile TiO_2_ in single H_2_ adsorbed surface was −1.43 eV and −1.45 eV, respectively, indicating the adsorption of H_2_ is more favorable with the sample with oxygen vacancy.

In the present work, we used a density functional theory with the Hubbard parameter (DFT + U) approach to investigate the effect of S doping and oxygen vacancy on the structural, electronic, mechanical, and optical properties of rutile TiO_2_ for photocatalysis application. The effects of metals and non-metals doping on the physical properties of anatase TiO_2_ are widely studied by both experimental and DFT approaches. However, to the best of our knowledge, there are limited studies on the effects of non-metals and oxygen vacancy on the physical properties of rutile TiO_2_ by the DFT + U method. Furthermore, there are limited studies on the effects of varying S concentration through DFT + U. Therefore, this study aimed to investigate the influence of S concentrations and oxygen vacancy on the physical properties of rutile TiO_2_. Our present findings are expected to guide the design of advanced TiO_2_-based materials for a range of applications, including photocatalysis and optoelectronic devices.

## 2. Computational Details

The spin-polarized density functional theory (DFT) using Quantum ESPRESSO [22] is performed to investigate the influence of S doping and oxygen vacancy on the structural, electronic, mechanical, and optical properties of rutile TiO_2_. The lattice parameters a = b = 4.59 Å and c = 2.96 Å, and angle parameters α = β = γ = 90°, are taken from experimental data [23]. The tetragonal crystal structure of rutile TiO_2_ is visualized by using XCrySDen [24]. The generalized gradient approximation (GGA) with the Perdew–Burke–Ernzerhof (PBE) functional was employed [25]. The ultrasoft pseudopotential is used in all calculations except in optical properties as it is not implemented for optical properties determinations. First, convergence tests were performed for the plane-wave cutoff energy, varying it from 15 Ry to 120 Ry, and the converged value was found to be 45 Ry. Then, k-points for the integration over the Brillouin zone were tested, with the converged 6 × 6 × 5 k-point grid being used in the calculations. A 2 × 2 × 2 supercell, containing 48 atoms (16 Ti atoms and 32 O atoms), was constructed using VESTA 3 software [26] to incorporate S doping. Full geometry optimization was performed for both undoped and doped rutile TiO_2_ using the Broyden–Fletcher–Goldfarb–Shanno (BFGS) minimizing method. The convergence criteria for total energy and forces were set to 10^−6^ eV and 10^−3^ eV/Å, respectively. The dopant site was selected based on the computed defect formation energies by substituting a single S atom at the Ti and O sites. In the case of Ti substitution, the formation energy was found to be 2.73 eV, while, for O substitution, the formation energy was −2.16 eV, indicating that S doping at the O site is more stable. The oxygen vacancy structure of rutile TiO_2_ was also optimized for the subsequent calculations. For the partial and total density of states, a dense k-point mesh of 18 × 18 × 15 was used. In the band structure calculations, the high symmetry points were applied. To improve the description of localized 3d electrons in rutile TiO_2_, the Hubbard U correction was applied to the Ti 3d orbitals, using a value of U = 7.5 eV. The inclusion of the Hubbard U term allows for a more accurate treatment of the strongly correlated Ti 3d states, which is expected to provide a better description of the electronic structure, such as the bandgap, the positions of impurity states, and the localization of charge carriers. This correction enhances the prediction of electronic properties, particularly for systems exhibiting strong correlation effects, such as oxygen vacancies and doped materials. Without adding Hubbard U correction, the Ti 3d states are treated within the standard DFT framework, without explicitly accounting for the on-site Coulomb repulsion. This typically leads to the over-delocalization of the 3d electrons, particularly in systems with transition metals, resulting in inaccuracies in the predicted electronic structure, such as bandgap underestimation and improper localization of the oxygen vacancies. For the mechanical properties, the thermo_pw code implemented in Quantum ESPRESSO was utilized. Finally, norm-conserving pseudopotentials were employed for the optical property investigations.

## 3. Results and Discussion

### 3.1. Structural Properties

**Figure 1:**
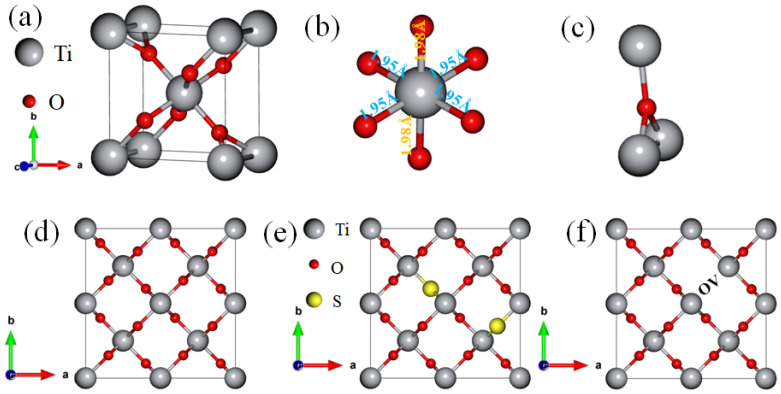
(**a**) Bulk structure of rutile TiO_2_, (**b**) octahedra TiO_6_, (**c**) Ti_3_O, (**d**) 2 × 2 × 2 supercell, (**e**) 6.25% S doped TiO_2_, (**f**) 2 × 2 × 2 TiO_2_ with oxygen vacancy.

Rutile TiO_2_ adopts a tetragonal crystal structure with the P42/mnm space group, as shown in Figure 1a. In this structure, a single Ti^4+^ ion is coordinated to six O^2−^ ions, forming TiO_6_ octahedra that share edges and corners, as depicted in Figure 1b. The structure features two distinct bond lengths: the shorter bond of 1.95 Å, which corresponds to four Ti-O bonds, and the longer bond of 1.98 Å, which corresponds to two Ti-O bonds. The O^2−^ ions are coordinated with three equivalent Ti4+ ions in a distorted trigonal geometry, as illustrated in Figure 1c. The crystallographic parameters, sourced from the Materials Project, include lattice constants a = b = 4.59 Å and c = 2.96 Å, with cell angles α = β = γ = 90° [23]. Upon constructing the supercell, the lattice constants are expanded to a = b = 9.2 Å and c = 5.92 Å. After performing full geometry optimization, the resulting lattice constants and cell volumes for both undoped and doped TiO_2_, as well as for the oxygen vacancy configurations, are presented in Table 1.

The lattice parameters of rutile TiO_2_, including the effects of S doping and OV, were calculated and compared with experimental data. The lattice constant obtained for undoped rutile TiO_2_ is in the range of both the reported experimental values and previous DFT studies. S doping was found to increase the lattice constant along all axes, indicating an expansion of the unit cell. This expansion can be attributed to the larger atomic radius of S compared to O [27,28]. In contrast, the introduction of OV caused a decrease in the lattice constant, likely due to defects resulting from oxygen deficiency. Specifically, the creation of oxygen vacancy led to a slight contraction of the lattice, which can be explained by the reduced local atomic density around the vacancy site.

Moreover, bond lengths and bond angles are crucial parameters for understanding the distortion or modification of the TiO_2_ lattice due to S doping and oxygen vacancy creation. The obtained bond length and bond angles during S doping and oxygen vacancy are presented in Table 1. In the case of S doping, as S has a larger atomic radius than O, the Ti-S bond length will generally increase compared to the Ti-O bond in undoped TiO_2_. This elongation of the bond length is due to the larger ionic radius of S^2−^ (1.84 Å) compared to O^2−^ (1.40 Å). Increasing the S doping concentration increased bond length, as presented in Table 1. The creation of an oxygen vacancy in the TiO_2_ structure also altered the bond lengths. Typically, the Ti-O bonds around the oxygen vacancy will adjust due to the loss of electron density associated with the missing oxygen atom. The Ti-O bond lengths near the vacancy tend to shorten as the Ti atom may undergo a slight contraction in its coordination environment.

On the other hand, bond angles can deviate from the ideal 90° when external elements and oxygen vacancy are inserted in the structure of materials. In the case of S doping, the larger atomic radius of S distorts the local geometry, changing the bond angles. As is shown in Table 1, the bond angles deviated from 90° due to S doping. The oxygen vacancy can significantly alter the coordination environment of the surrounding Ti atoms. As Ti atoms adjust to the missing oxygen, the bond angles might deviate from the typical geometry of undoped TiO_2_. The bond angle is shifted to the lowest point due to the missing O atoms, as presented in Table 1.

**Table 1:** The obtained lattice constants and cell volume from full geometry optimizations.

Materials	Lattice Constant (Å)		Vol. (Å)^3^	Bond Angle (Å)	Bond Length (°)
	a = b	c			
Undoped rutile TiO_2_	4.63	2.98	63.7	Ti-O = 1.9518 Ti-O = 1.9796	Ti-O-Ti = 90
Expt. work [29]	4.61	2.96	62.91	Ti-O = 1.9500 Ti-O = 1.9800	--
Other DFT [17,30]	4.68	2.98	--	Ti-O = 1.961	--
6.25% S doped	4.89	3.27	73.3	Ti-S = 2.0080 Ti-S = 2.1050	Ti-S-Ti = 106.6
12.5% S doped	5.14	3.69	80.4	Ti-S = 2.0497 Ti-S = 2.4021	Ti-S-Ti = 113.2
18.75% S doped	5.31	3.82	93.3	Ti-S = 2.0551 Ti-S = 2.6113	Ti-S-Ti = 111.4
OV	4.17	2.82	53.8	Ti-S = 1.5887 Ti-S = 1.8430	Ti-O-Ti = 87.9

**Figure 2:**
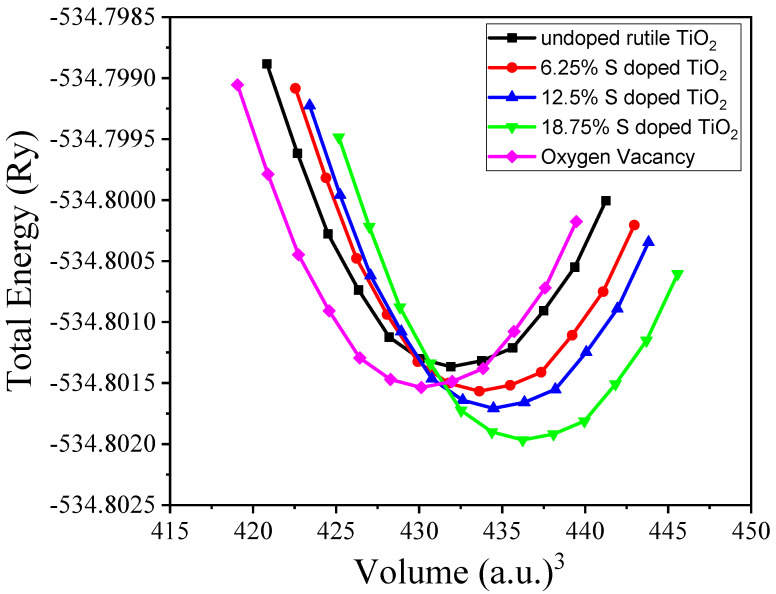
Total energy versus volume at equilibrium.

The equilibrium volume (V_0_), bulk modulus (B_0_), and pressure derivatives of the bulk modulus (dB_0_) were calculated using the Murnaghan equation of state [31], as implemented in Quantum ESPRESSO. The total energy as a function of volume, which was used to determine the equilibrium properties, is shown in Figure 2. The obtained values for the equilibrium volume, bulk modulus, and pressure derivatives are provided in Table 2. These results were compared with previous reports and are in good agreement with both the experimental data and other DFT studies. When S concentrations increase, all equilibrium values increase, which may be attributed to the local geometry distortion due to S doping. In contrast, in the presence of oxygen vacancy, the equilibrium volume and bulk modulus showed a decrease compared to the undoped TiO_2_ due to the lack of oxygen in the structure. The discrepancies observed between the present work and the other DFT calculations in Table 2 could be partly attributed to differences in the choice of exchange-correlation (XC) functionals, as well as other computational parameters such as the k-point sampling, supercell size, and basis set.

Furthermore, formation energies of S atoms incorporated into the Ti and O sites are calculated to better understand the stability and energetics of these doped systems. The formation energy for the S-doped rutile TiO_2_ is calculated by using Equation (1) [32]:

(1) $$E_{f}=E_{S-doped\;{TiO}_{2}}-E_{undoped\;TiO_{2}}-n(\mu_{S}-\mu_{O})$$

where $E_{f}$ is formation energy, $E_{S-doped\;{TiO}_{2}}$ is total energy of S doped, *n* is number of dopant atoms, and $\mu_{S}$ and $\mu_{O}$ are the chemical potentials of the S and O atoms, which can be obtained by DFT total energies calculations of ground states [33]. In this calculation, we assume the Ti-rich condition because the dopants are substituted at O sites and reduce the number of O atoms in the lattice.

**Table 2:** The equilibrium V_0_, B_0_, and dB_0_ of undoped TiO_2_, S doped, and oxygen vacancy.

Sources	V_0_	B_0_ (GPa)	dB_0_
Present work	63.7	229.5	4.04
Exp. work [34]	62.5	212	6.3
Other DFT [35]	64.03	235	4.64
6.25% S doped	64.4	231.3	5.69
12.5% S doped	64.6	301.9	5.81
18.75% S doped	65.2	305.2	5.90
OV	62.4	207.6	3.15

The calculated results of formation energy in substituting S at Ti and O sites, varying S concentrations, and TiO_2_ with oxygen vacancy are presented in Table 3. The formation energy of S doped at Ti sites is typically higher compared to doping at O sites, reflecting the inherent differences in the local environment and bond strengths at these sites. Doping S at Ti sites is energetically less favorable, requiring higher energy input. On the other hand, S atoms replacing O atoms can effectively occupy the O sites, with relatively lower formation energy, suggesting that this type of doping is more thermodynamically stable. As the concentration of S increases, the formation energy also increases. This observation can be attributed to the lattice strain and the increasing repulsion between dopant atoms at higher concentrations, which makes the system less energetically favorable [32]. However, despite the increase in formation energy with higher doping concentrations, the system remains thermodynamically stable, as evidenced by the negative formation energies. Among all S concentrations considered in this study, the 6.25% sulfur doping concentration stands out with the lowest formation energy, indicating that this particular concentration is the most thermodynamically favorable.

### 3.2. Electronic Properties

#### 3.2.1. Band Structures

**Figure 3:**
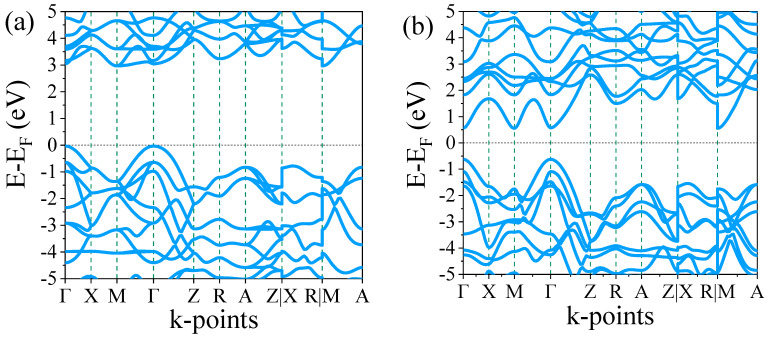

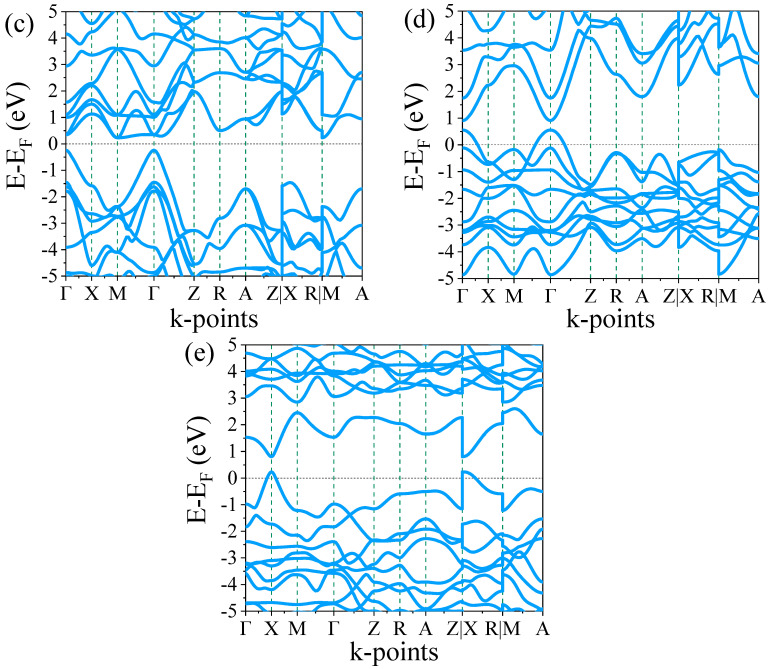
Electronic band structures of (**a**) undoped TiO_2_, (**b**) 6.25% doped, (**c**) 12.5% doped, (**d**) 18.75% doped, and (**e**) TiO_2_ with oxygen vacancy.

The electronic structure of undoped and S-doped TiO_2_, as well as oxygen vacancy systems, was investigated by calculating the electronic band structure and density of states (DOS). The computed band structures for undoped, S-doped TiO_2,_ and oxygen vacancy systems are presented in Figure 3a–e. The band structure calculations were performed along the high symmetry path in the Brillouin zone (BZ) Γ—X—M—Γ—Z—R—A—Z|X—R|M—A. In the plot of band structures and density of states, the Fermi energy level is set to 0 eV. As shown in Figure 3a, the undoped rutile TiO_2_ exhibits a direct bandgap of 3.01 eV, which limits its photocatalytic activity under visible light. Thus, both conduction band minimum (CBM) and valence band maximum (VBM) are found at the Γ high symmetry point. The bandgap obtained for undoped rutile TiO_2_ in our study is consistent with experimentally reported values [36]. This accurate result can be attributed to the inclusion of the Hubbard correction for the Ti 3d orbitals, which enhances the treatment of the electron–electron interactions and ensures a more realistic description of the electronic structure in TiO_2_.

S doping introduces new states within the bandgap, resulting in a significant reduction in the bandgap, which enables TiO_2_ to absorb visible light. For the 6.25% S doping, the sulfur-induced states are positioned just below the CBM, facilitating electronic transitions under visible light excitation. As shown in Figure 3b, for 6.25% S doping, the CBM and VBM occur at the Γ point, similar to the undoped rutile TiO_2_, but with a significant reduction in the bandgap to 1.3 eV. This reduction in bandgap can be attributed to the expansion of the unit cell when sulfur occupies oxygen sites, due to the difference in atomic radii [27]. As discussed in the structural properties section, the lattice constants increase significantly with S doping, which may also contribute to the observed decrease in the bandgap. For 12.5% S doping, the position of the CBM shifts to the M high symmetry point, while the VBM remains at the Γ point, as shown in Figure 3c, resulting in an indirect bandgap of 0.67 eV. With 18.75% S doping, the bandgap is further reduced to a direct bandgap of 0.4 eV, as can be seen from Figure 3d. Generally, increasing the S concentration reduced the bandgap significantly because increasing dopant concentrations introduces extra energy levels within the bandgap, effectively narrowing it. This trend is consistent with experimental findings reported by Shah et al. [37], who observed similar bandgap narrowing with increasing dopant concentrations. In their study, Mn doping in ZnO resulted in a bandgap reduction from 3.3 eV to 2.75 eV as the Mn concentration increased. Both experimental and computational studies have consistently shown that S doping tends to narrow the bandgap of materials when incorporated into host structures [38,39,40].

On the other hand, introducing oxygen vacancy (OV) in rutile TiO_2_ significantly alters its electronic structure and surface reactivity. The presence of oxygen vacancy introduces localized energy states within the bandgap, leading to enhanced electrical conductivity by facilitating charge carrier mobility. The electronic band structure of oxygen vacancy in rutile TiO_2_ is shown in Figure 3e. It shows a direct bandgap of 0.6 eV occurring at X points. Similarly, bandgap reduction through oxygen vacancy in rutile TiO_2_ was reported by Guan et al. [41]. Oxygen vacancies can act as electron donors, increasing the concentration of free electrons, which can be beneficial in photocatalytic reactions and in improving the material’s performance in energy-related applications like supercapacitors and lithium-ion batteries. Furthermore, oxygen vacancies enhance the photocatalytic efficiency of rutile TiO_2_ by increasing its ability to generate reactive oxygen species (ROS) under UV light irradiation [7]. These defects are also known to modify the band alignment and reduce the recombination rates of electron–hole pairs, thus improving the material’s photocatalytic performance.

#### 3.2.2. Density of States

**Figure 4:**
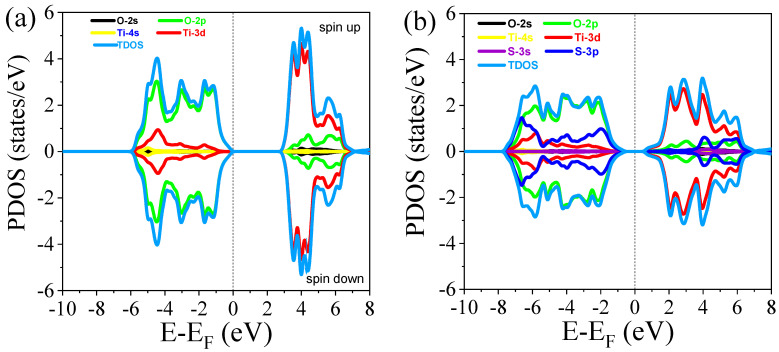

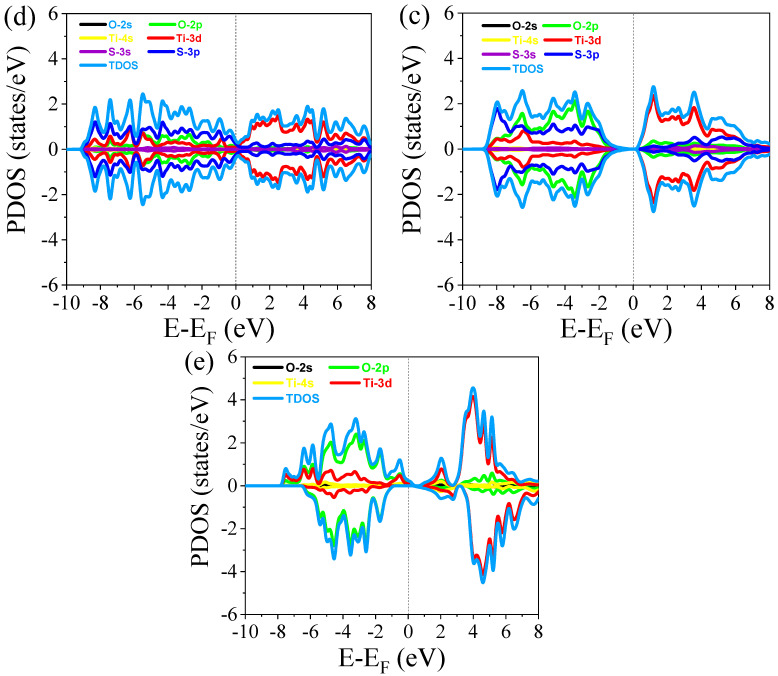
Partial density of states (PDOS) of (**a**) undoped TiO_2_, (**b**) 6.25% doped, (**c**) 12.5% doped, (**d**) 18.75% doped, and (**e**) TiO_2_ with oxygen vacancy.

The partial density of states (PDOS) and total density of states (TDOS) for both undoped and S-doped rutile TiO_2_, as well as oxygen vacancies in TiO_2_, are shown in Figure 4a–e. For undoped TiO_2_, the Fermi energy level is at the edge of the valence band maximum (VBM), as shown in Figure 4a, indicating that undoped rutile TiO_2_ exhibits p-type conductivity characteristics. The PDOS illustrates the contribution of each element’s orbitals to the states, with the valence band primarily composed of O 2p orbital contributions. The conduction band is formed mainly by Ti 3d orbitals, suggesting that the bond between Ti and O is ionic in nature. In this case, the bandgap value is 3.01 eV, which aligns with the results from the band structure analysis. The spin-polarized calculations reveal symmetric features, indicating that rutile TiO_2_ is a non-magnetic material.

For the 6.25% S doping, the PDOS shows the contribution of the S dopant orbitals in both the conduction and valence bands, with the major contribution occurring in the valence band. As observed in Figure 4b, the effect of S doping is more pronounced at the edge of the valence band, whereas its presence in the conduction band is farther from the edge. The incorporation of S in the rutile TiO_2_ lattice is supported by the contribution of the S 2p orbital. The Fermi level shifts toward the conduction band, transforming the material’s p-type behavior into n-type behavior. At 12.5% S doping, the bandgap narrows to 0.67 eV, and the Fermi level shifts even further into the conduction band compared to the 6.25% doping. In all concentrations of S doping, the effect of S doping is more prominent at the edges of the valence band, though it also contributes to the conduction band, as shown in Figure 4b–d. As the concentration of S increases, the number of states increases, which is evident from the emergence of peaks in the density of states.

Unlike S doping, the PDOS and TDOS of oxygen vacancy in rutile TiO_2_ exhibit an asymmetric distribution, as shown in Figure 4e. This asymmetry indicates that the introduction of oxygen vacancy induces magnetic behavior in the host material, rutile TiO_2_. Similar to the undoped rutile TiO_2_, the valence band is primarily composed of O 2p orbital contributions, while the conduction band is dominated by Ti 3d orbital contributions. However, the presence of oxygen vacancies results in a significant change in the material’s electronic structure. Specifically, the oxygen vacancies introduce a mid-gap state, which narrows the bandgap and shifts the Fermi level. This modification enhances the material’s response to visible light, as the defect states created by oxygen vacancies contribute to the absorption of lower-energy photons, thus improving the photocatalytic properties of the material. Additionally, the oxygen vacancy-induced magnetic behavior further distinguishes OV-doped rutile TiO_2_ from its undoped counterpart, making it a promising candidate for a range of advanced applications.

### 3.3. Mechanical Properties

The mechanical properties of undoped TiO_2_, S-doped TiO_2_, and TiO_2_ with oxygen vacancy in rutile TiO_2_ were evaluated by calculating the elastic constants (*C_ij_*) by using thermo_pw code implemented in Quantum ESPRESSO. Thus, the elastic constant can be calculated by Taylor expansion of the total energy [42] which is given as follows:

(2) $$E\left(V,\epsilon \right)=E\left(V_{0},0\right)+V_{0}\sum_{i}\tau_{i}\gamma_{i}\epsilon_{i}+\frac{1}{2}V_{0}\sum_{ij}C_{ij}{\epsilon_{i}\tau }_{ij}\gamma_{i}\epsilon_{j}\gamma_{j}$$

where $E\left(V,\epsilon \right)$ is the Taylor expansion total energy, $E\left(V_{0},0\right)$ is the unstrained energy with a volume at equilibrium, $\tau_{i}$ are the stress tensor elements, $\gamma_{i}$ is the factor for the Voigt index, and $C_{ij}$ are the elastic constant components. The elastic constants have a 6 × 6 matrix with 27 different components. According to the symmetry of tetragonal rutile TiO_2_, the components are reduced to six elements, including *C*_11_, *C*_12_, *C*_13_, *C*_33_, *C*_44_, and *C*_66_ [43]. For the elastic constant, the tetragonal structure stability according to the Born stability conditions is as [44] follows: (*C*_11_ − *C*_12_) > 0, *C*_11_ > 0, *C*_33_ > 0, (*C*_11_ + *C*_33_ − 2*C*_13_) > 0, (2*C*_11_ + *C*_33_ + 2*C*_12_ + 4*C*_13_) > 0, *C*_44_ > 0, and *C*_66_ > 0. Our calculated result satisfies these conditions for rutile TiO_2_, indicating its phase stability.

The computed elastic constants for undoped rutile TiO_2_ are in good agreement with previous experimental and DFT works except for a small deviation, which can be attributed to the different conditions or parameters that were used. The comparisons of the obtained elastic constants with different reported values are presented in Table 4. Upon introducing S doping, we observe a slight increase in the elastic constants, indicating a marginal enhancement in the material’s stiffness. This could be attributed to the stronger Ti–S bonding interactions when S substitutes at O sites, which reinforces the overall structural integrity. In contrast, oxygen vacancy leads to a noticeable decrease in the elastic constants. The reduction in stiffness is linked to the local structural distortions and weaker bonding associated with the missing oxygen atoms, which reduces the overall resistance to deformation. However, the system remains mechanically stable, as indicated by the positive values of the elastic constants.

Other mechanical properties, including bulk modulus, shear modulus, and Young’s, modulus can be calculated by using the obtained elastic constants. These mechanical properties of materials provide crucial insights into their response to external stresses. The bulk modulus and shear modulus according to the Voigt approximation [45] is calculated by Equations (3) and (4), respectively.

(3) $$B_{V}=0.11\left(2C_{11}+2C_{12}\right)+4C_{13}+C_{33}$$

and

(4) $$S_{V}=0.07(2C_{11}-C_{12}-2C_{13}+C_{33}+4C_{44}+3C_{66})$$

The single crystal elastic constants (*S_ij_*) are used to calculate the bulk modulus and shear modulus in the Reuss approximation. Thus, the *S_ij_* for six elements can be derived from the elastic constant (*C_ij_*) as follows:

(5) $$S_{11}=\frac{C_{11}C_{33}-C_{13}^{2}}{\left(C_{11}-C_{12}\right)C_{33}\left(C_{11}+C_{12}\right)-2C_{13}^{2}}$$

(6) $$S_{12}=\frac{-C_{12}C_{33}+C_{13}^{2}}{\left(C_{11}-C_{12}\right)C_{33}\left(C_{11}+C_{12}\right)-2C_{13}^{2}}$$

(7) $$S_{13}=\frac{-C_{13}}{C_{33}\left(C_{11}+C_{12}\right)-2C_{13}^{2}}$$

(8) $$S_{33}=\frac{C_{11}+C_{12}}{C_{33}\left(C_{11}+C_{12})-2C_{13}^{2}\;\right)}$$

(9) $$S_{44}=\frac{1}{C_{44}}$$

(10) $$S_{66}=\frac{1}{C_{66}}$$

The bulk modulus (*B_R_*) and shear modulus (*S_R_*) can be derived by the Reuss approximation [46] as follows:

(11) $$B_{R}=\frac{1}{2(S_{11}+S_{12}{+2S}_{13}+S_{33})}$$

(12) $$S_{R}=\frac{1}{2\left(4S_{11}-2S_{12}{-4S}_{13}+2S_{33}\right)+3(2S_{44}+S_{66})}$$

The bulk modulus (*B_H_*) quantifies the material’s resistance to uniform compression and is an important indicator of the material’s overall stability under pressure. The Hill approximation used the average of the Voigt and Reuss approximations to calculate the bulk modulus (*B_H_*) and shear modulus (*S_H_*).

(13) $$B_{H}=0.5(B_{V}+B_{R})$$

and

(14) $$S_{H}=0.5(S_{V}+S_{R})\;$$

The calculated bulk modulus of undoped TiO_2_, S-doped, and TiO_2_ with oxygen vacancy is presented in Table 4. For S-doped rutile TiO_2_, the DFT calculations show a slight increase in the bulk modulus compared to the undoped TiO_2_. The substitution of S atoms in the TiO_2_ lattice creates local distortions in the crystal structure, which may strengthen the atomic interactions and lead to an increase in the material’s resistance to compression. In contrast, for TiO_2_ with oxygen vacancies, DFT predicts a slight decrease in the bulk modulus relative to the undoped TiO_2_. The oxygen vacancies alter the Ti-O bonding network, causing local structural rearrangements that can lead to it being more compact and that may distort the lattice in certain regions of the material, thus reducing its resistance to uniform compression.

The shear modulus measures a material’s resistance to shear deformation, providing an understanding of its rigidity when subjected to tangential forces. In S-doped rutile TiO_2_, the DFT results indicate an increase in the shear modulus, which is consistent with the observed increase in bond strength due to S doping. For TiO_2_ with oxygen vacancies, DFT predicts a decrease in the shear modulus, which suggests a reduction resistance to shear deformation.

Young’s modulus is a measure of a material’s overall stiffness under tensile stress and provides an indication of its ability to resist elongation. Young’s modulus (Y) can also be derived from Hill approximation as follows:

(15) $$Y=\frac{9B_{H}S_{H}}{3B_{H}+S_{H}}$$

The calculated young’s modulus is given in Table 4. DFT calculations show that S-doped rutile TiO_2_ exhibits a higher Young’s modulus compared to undoped TiO_2_, which increases the overall stiffness of the material. In the case of TiO_2_ with oxygen vacancies, DFT predicts a reduction in Young’s modulus, which is more significant compared to undoped TiO_2_. The oxygen vacancy creates defects in the TiO_2_ lattice, weakening the material’s overall resistance to elastic deformation. The vacancies disrupt the regular Ti-O bonding network, which reduces the structural rigidity of the material and results in a softer response to applied tensile stress.

The value of Poisson’s ratio (*n*) can also be calculated by the following:

(16) $$n=0.5\frac{3B_{H}-2S_{H}}{3B_{H}+S_{H}}$$

The Poisson’s ratio provides insight into the ductility of materials. As shown in Table 4, the Poisson’s ratio for undoped rutile TiO_2_ is 0.24, indicating relatively low ductility. However, when S is doped into the material, the ductility improves. In contrast, the presence of oxygen vacancies leads to a reduction in ductility, likely due to the absence of the S dopant.

### 3.4. Optical Properties

The optical properties of materials are crucial for understanding their behavior in various applications such as photocatalysis, solar cells, and optoelectronic devices. To examine the optical properties, we computed the dielectric function, which consists of three components: *ε_xx_*(*ω*), *ε_yy_*(*ω*), and *ε_zz_*(*ω*). For all optical calculations, the average of these three components was taken [47]. Since our calculations include spin-polarized data, for undoped TiO_2_, S-doped, and TiO_2_ with oxygen vacancy, the spin-up and spin-down state data were combined to produce a single optical plot for each system.

#### 3.4.1. Real Part of Dielectric

The complex dielectric function is an important quantity that provides insight into a material’s polarization response to an applied electric field, including its interaction with electromagnetic waves and is given by the following [47,48]:

(17) $$\varepsilon \left(\omega \right)=\varepsilon_{1}\left(\omega \right)+i\varepsilon_{2}\left(\omega \right)$$

where $\varepsilon_{1}$ is a real dielectric and $\varepsilon_{2}$ is an imaginary dielectric function.

**Figure 5:**
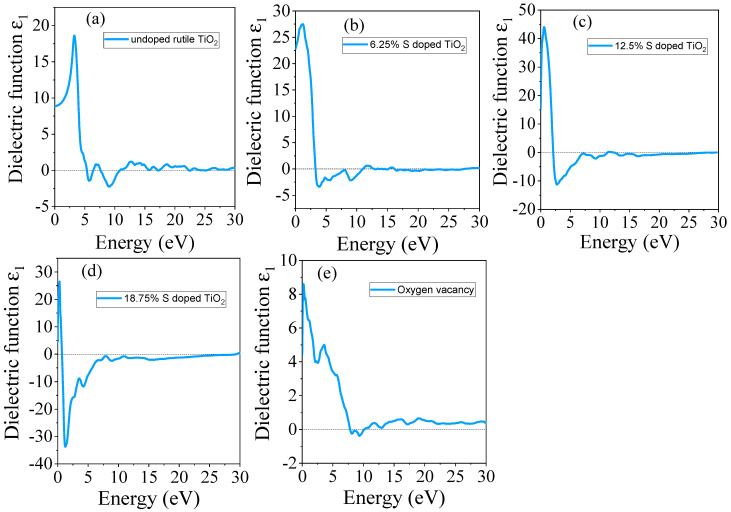
The computed real part of the dielectric function of (**a**) undoped TiO_2_, (**b**) 6.25% doped, (**c**) 12.5% doped, (**d**) 18.75% doped, and (**e**) TiO_2_ with oxygen vacancy.

The real and imaginary parts of the dielectric function were computed for each configuration (undoped TiO_2_, S-doped TiO_2_, and TiO_2_ with oxygen vacancies) across the photon energies from 0 to 30 eV. The obtained real dielectric function of undoped TiO_2_, S-doped, and TiO_2_ with oxygen vacancy is presented in Figure 5a–e. The dielectric function of undoped TiO_2_ shows a sharp increase in the real part (*ε*_1_) in the UV region, corresponding to the onset of interband transitions, as shown in Figure 5a. Negative values of the real part of the dielectric function are observed as the photon energy increases into the ultraviolet (UV) range, suggesting that the material exhibits a high amount of reflection of incoming electromagnetic radiation and limited transmission. For undoped TiO_2_, the maximum peak of the real dielectric function is revealed at 3.27 eV.

The doping of S atoms into TiO_2_ results in a significant shift in the maximum peak real dielectric function. Thus, the real part of the dielectric function in the visible range increases, which aligns with the improved absorption properties as shown in Figure 5b–d. For the 18.75% doped material, the negative value of the real part of the dielectric function increased, indicating high reflectivity due to the narrowing of the bandgap, which gives the material metallic properties. For the real part of the dielectric function, the peaks broaden to visible light with increasing S dopant concentrations. On the other hand, the introduction of oxygen vacancy leads to a noticeable change in the dielectric function. The real dielectric function of TiO_2_ with oxygen vacancy is shown in Figure 5e. The absorption features near the band edge become more pronounced, and the real part of the dielectric function is increased at lower photon energies, indicating that oxygen vacancy promotes absorption in the visible light range. The calculated real dielectric values at static (0 eV) are given in Table 5. The positive values of the real dielectric function at static (*ε*_1_) (0) in the visible light range suggest that the material exhibits low reflection, indicating favorable optical properties and transparency [49].

**Figure 6:**
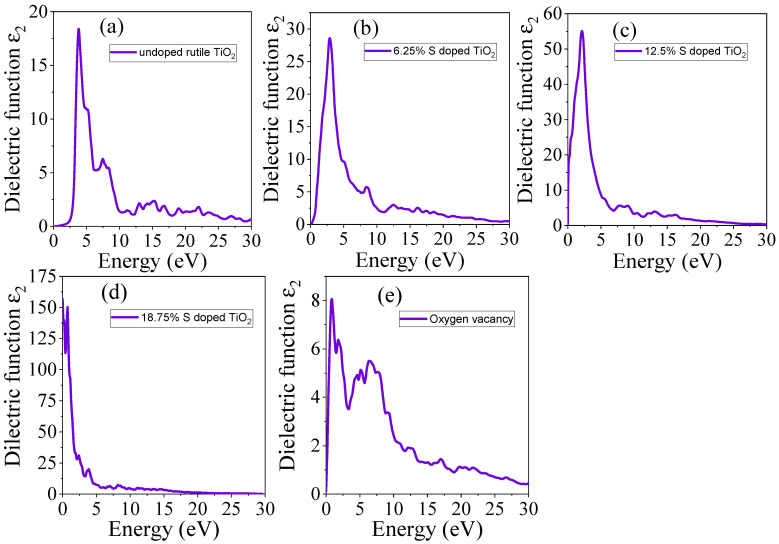
The computed imaginary part of dielectric function of (**a**) undoped TiO_2_, (**b**) 6.25% doped, (**c**) 12.5% doped, (**d**) 18.75% doped, and (**e**) TiO_2_ with oxygen vacancy.

The imaginary part of the dielectric function represents the material’s response to electromagnetic fields, specifically related to energy absorption [50]. The obtained imaginary dielectric function of undoped TiO_2_, S-doped, and TiO_2_ with oxygen vacancy is presented in Figure 6a–e. For undoped TiO_2_, the imaginary part (*ε*_2_) peaks at the energy corresponding to the bandgap, confirming the material’s high absorption in the UV range, as shown in Figure 6a. After S doping, the imaginary part broadens indicating that S doping enhances the material’s ability to absorb light over a wider range of photon energies, as shown in Figure 6b–d. A high imaginary part of the dielectric function usually indicates strong absorption, while a low value suggests weaker absorption. As shown in Figure 6b–d, the dielectric value increases with higher concentrations of the S dopant. However, in the presence of oxygen vacancies, the peak of the imaginary part decreases, although it shifts to the visible region compared to pure TiO_2_.

#### 3.4.2. Refractive Index

The refractive index (*n*) was also calculated to provide further insights into the optical behavior of the material. The refractive index determines the speed of light in the material. The refractive index is directly related to the real part of the dielectric function and can be calculated by Equation (18) [51]:

(18) nω=0.707ε12ω+ε22ω+ε1(ω)12

**Figure 7:**
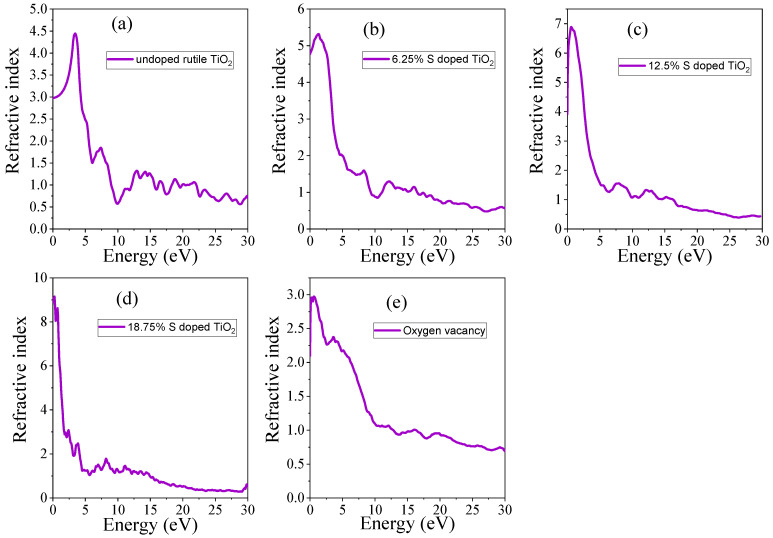
The computed refractive index of (**a**) undoped TiO_2_, (**b**) 6.25% doped, (**c**) 12.5% doped, (**d**) 18.75% doped, and (**e**) TiO_2_ with oxygen vacancy.

The calculated refractive index of undoped TiO_2_ shows typical values for a wide bandgap material, with a higher refractive index in the UV region, as shown in Figure 7a. The S doping impact on the refractive index can be observed from Figure 7b–d. Sulfur doping results in a lower refractive index compared to undoped TiO_2_ in the UV region, reflecting the decrease in the material’s band gap. However, in the visible region, each concentration has a higher refractive index than the undoped TiO_2_. Thus, in doped materials, the light can stay for long periods of time, which can enhance the photocatalytic activity of the materials. The impact of oxygen vacancy on the refractive index can be observed in Figure 7e. Oxygen vacancy leads to a shift in the maximum refractive index to the visible region, as a result of the creation of additional electronic states that facilitate light absorption. The static refractive index of undoped TiO_2_, doped TiO_2_, and TiO_2_ with oxygen vacancy is provided in Table 5. At static conditions (0 eV), the refractive index increases as the concentration of the S dopant increases, indicating enhanced absorption of visible light. This suggests that doping with S improves the material’s ability to interact with visible light, potentially making it more effective for applications such as photocatalysis or optoelectronics.

#### 3.4.3. Absorption Coefficient

The absorption spectrum of a material determines the wavelengths of light it can absorb. The optical absorption coefficient (*α*) can be calculated by the following [51]:

(19) $$\alpha \left(\omega \right)=\frac{4\pi k\left(\omega \right)}{\lambda }=\frac{4\pi }{hc}(kE)$$

where *λ* is the wavelength, *k* is the extinction coefficient, *h* is Plank’s constant, *c* is the speed of light, and *E* is the photon energy.

**Figure 8:**
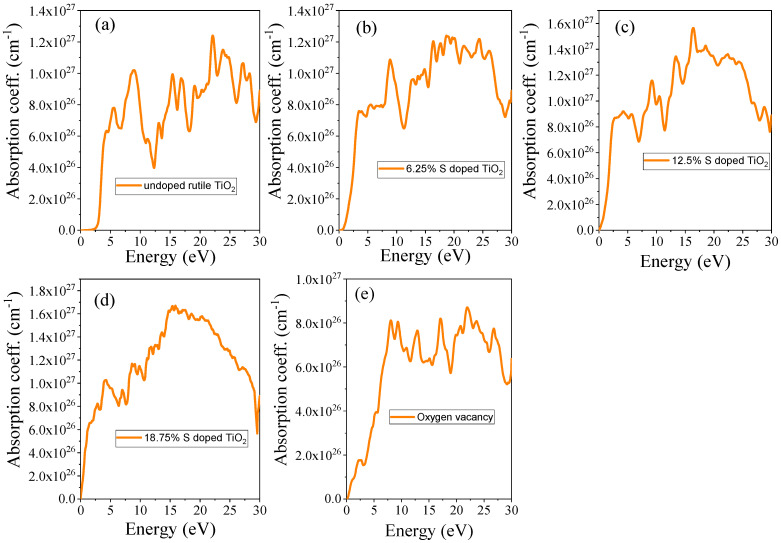
The refractive index of (**a**) undoped TiO_2_, (**b**) 6.25% doped, (**c**) 12.5% doped, (**d**) 18.75% doped, and (**e**) TiO_2_ with oxygen vacancy.

In this study, the absorption coefficient (*α*) of undoped rutile TiO_2_, S-doped TiO_2_, and TiO_2_ with oxygen vacancy was calculated over a broad range of photon energies from 0 to 30 eV and is presented in Figure 8. The result shows that S doping and oxygen vacancy significantly enhance the optical absorption in the visible regions of the spectrum compared to undoped TiO_2_. In its pristine form, TiO_2_ exhibits a significant absorption onset in the ultraviolet (UV) region due to its wide band gap of ~3.01 eV. However, its absorption in the visible light range is limited, as shown in Figure 8a. Upon introducing S atoms into the TiO_2_ lattice, the absorption spectrum shifts towards the visible region, as can be seen in Figure 8b–d. This is attributed to the narrowing of the band gap caused by the substitution of O by S, which introduces states within the band gap, enhancing visible light absorption. As the concentration of sulfur (S) increases from 6.25% to 18.75%, the absorption edge extends further into the infrared region. This shift indicates a broadening of the material’s optical absorption spectrum, which suggests that higher sulfur doping enhances the material’s ability to absorb a wider range of electromagnetic radiation, including in the infrared. Such an effect could be beneficial for applications that require enhanced absorption across a broad spectrum, such as in solar energy harvesting or infrared sensing.

The absorption coefficient spectrum of TiO_2_ with oxygen vacancies is shown in Figure 8e. The presence of oxygen vacancies in TiO_2_ creates localized states in the mid-gap, as shown in the electronic band structures plot, which contribute to improved absorption in the visible range. These energy levels, introduced by the vacancies, function as shallow donor levels, promoting electron transitions that occur at lower photon energies. As a result, the material demonstrates enhanced absorption capabilities. In general, these materials exhibit a high absorption coefficient, as presented in Table 5. This enhanced absorption is advantageous for applications such as photocatalysis and solar energy devices, where the ability to absorb light efficiently is crucial.

#### 3.4.4. Reflectance

The reflectance (*R*) was calculated using the relation between the real and imaginary parts of the dielectric function, as the reflectance is closely linked to the material’s ability to absorb and transmit light. It can be calculated by Equation (20):

(20) $$R(\omega )=\frac{1+\kappa^{2}-2n+n^{2}}{1+\kappa^{2}+2n+n^{2}}$$

**Figure 9:**
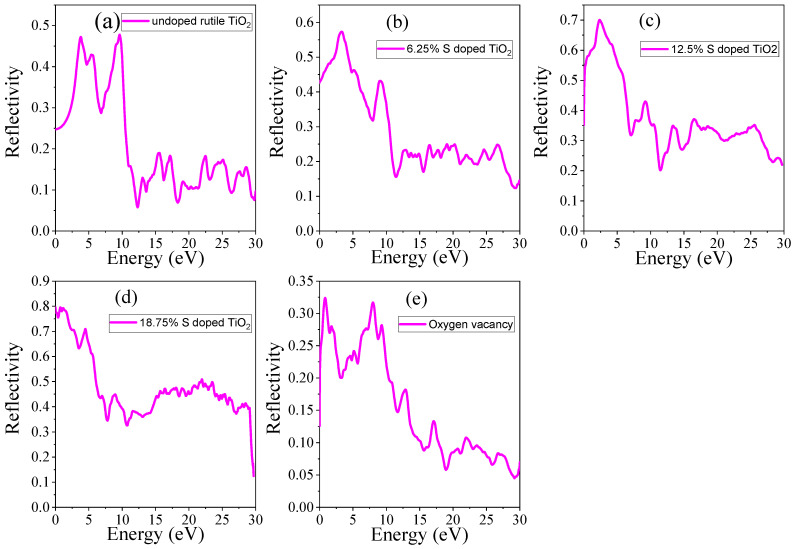
Reflectance of (**a**) undoped TiO_2_, (**b**) 6.25% doped, (**c**) 12.5% doped, (**d**) 18.75% doped, and (**e**) TiO_2_ with oxygen vacancy.

The computed reflectance spectra for undoped TiO_2_, S-doped TiO_2_, and TiO_2_ with oxygen vacancy are presented in Figure 9. In undoped TiO_2_, the static reflectance is 0.25, corresponding to 25% reflectance at 0 eV and a high reflectance of 46% is observed at 3.7 eV. This indicates that reflectance occurs at energy levels where light interacts with the material. In S-doped TiO_2_ and TiO_2_ with oxygen vacancy, the reflectance is observed at positions where the materials significantly interact with light. For the S-doped TiO_2_ with concentrations of 6.25%, 12.5%, and 18.75%, as well as TiO_2_ with oxygen vacancy, the static reflectance values are 42%, 35%, 79%, and 12%, respectively, as shown in Table 5. In the case of 18.75% S-doped TiO_2_, the static reflectance is particularly high due to a significantly narrowed bandgap, causing the material to behave more like a metal. As seen in Figure 9b–d, reflectance increases with doping compared to undoped TiO_2_. Similar findings have recently been reported by Badawi et al. [43] using an experimental method on Co-doped TiO_2_/FTO heterojunctions. However, in the presence of oxygen vacancy, the reflectance spectrum shows a noticeable decrease in the visible region, indicating increased absorption of visible light. The material still exhibits relatively high reflectance in the UV region, although this is slightly reduced compared to pristine TiO_2_ due to the presence of localized states that absorb lower-energy photons.

#### 3.4.5. Electron Energy Loss Spectrum (EELS)

The Electron Energy Loss Spectrum (EELS) provides crucial insights into the electron excitation from valence band to conduction band states and it can be calculated by Equation (21):

(21) $$EELS\left(\omega \right)=\frac{\varepsilon_{2}^{2}(\omega )}{\varepsilon_{2}^{2}\left(\omega \right)+\varepsilon_{1}^{2}\left(\omega \right)}$$

**Figure 10:**
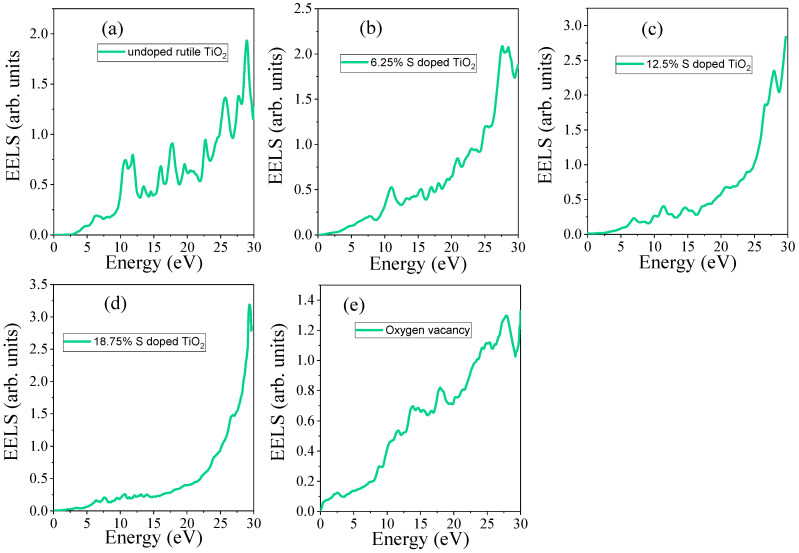
Electron energy loss spectrum (EELS) of (**a**) undoped TiO_2_, (**b**) 6.25% doped, (**c**) 12.5% doped, (**d**) 18.75% doped, and (**e**) TiO_2_ with oxygen vacancy.

EELSs of undoped TiO_2_, S-doped, and TiO_2_ with oxygen vacancy are shown in Figure 10. As shown in Figure 10a, for undoped TiO_2_, the EELS peak is not observed below 3.01 eV. S doping in TiO_2_ introduces sulfur-related states within the bandgap. In the EELS spectrum, this would manifest as new peaks corresponding to S 3p states, and the electronic transitions may shift due to the hybridization between S and O orbitals in the TiO_2_ lattice. As shown in Figure 10b–d, the emerged EEL spectrum is shifted to a lower photon energy as the dopant concentration increases.

On the other hand, oxygen vacancy introduces localized states in the bandgap, which also influences the EELS spectrum. This is typically observed as a shift and broadening of the peak, indicating changes in the bonding and the creation of new electronic states associated with the vacancy, as shown in Figure 10e. The maximum value of the EEL of the studied material is presented in Table 5. The EELS increases during S doping, and it is increased with increasing S concentrations. In contrast, in the presence of O vacancy, it is decreased due to the absence of S states.

#### 3.4.6. Optical Conductivity

The optical conductivity (σ) provides valuable insights into the electronic response of a material under an external electromagnetic field, revealing information about interband transitions and charge carrier dynamics. The optical conductivity of rutile TiO_2_ with S doping and oxygen vacancy is calculated to investigate how these modifications affect the material’s optical properties by using Equation (22).

(22) $$\frac{1}{4\pi }\left(\alpha \left(\omega \right)n\left(\omega \right)c\right)$$

**Figure 11:**
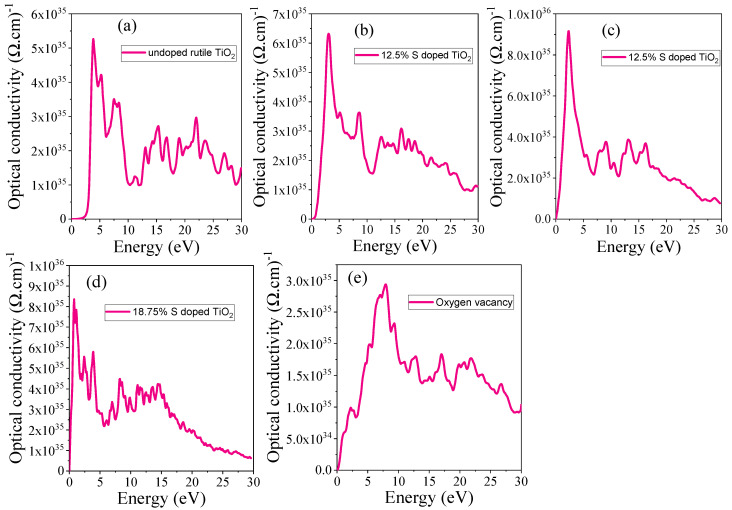
Optical conductivity of (**a**) undoped TiO_2_, (**b**) 6.25% doped, (**c**) 12.5% doped, (**d**) 18.75% doped, and (**e**) TiO_2_ with oxygen vacancy.

For undoped TiO_2_, the optical conductivity exhibits a sharp increase at 3.7 eV photon energies, corresponding to interband transitions from the valence band to the conduction band, with a characteristic onset related to the bandgap. Upon introducing S doping, the optical conductivity spectrum shows a noticeable shift, as shown in Figure 11b–d, indicating a modification of the electronic structure. The S-induced states in the conduction band lead to enhanced low-energy absorption, suggesting that S doping reduces the effective bandgap, which can enhance optical absorption in the visible light range. The maximum values of the obtained optical conductivity are listed in Table 5. After S doping, the values increased and shifted toward visible light, which can be attributed to the reduced bandgap due to the S states.

The presence of oxygen vacancy further alters the optical conductivity by introducing mid-gap states, as can be seen from the electronic structure sections. These localized states enable transitions at lower energies, leading to additional absorption features in the infrared and visible regions. The impact of S doping and oxygen vacancy results in a broader spectral response, with enhanced low-energy absorption, making TiO_2_ more suitable for applications that require visible light absorption, such as photocatalysis and solar energy conversion.

### 3.5. Photocatalytic Mechanisms

Sulfur doping in rutile TiO_2_ introduces S atoms into the crystal lattice, replacing some oxygen atoms. This doping creates new energy levels within the bandgap of rutile TiO_2_. These intermediate energy states act as electron donors, reducing the bandgap energy, which allows TiO_2_ to absorb visible light more efficiently [52,53]. This absorption shift facilitates the activation of the photocatalyst under visible light, unlike undoped rutile TiO_2_, which primarily absorbs UV light.

**Figure 12:**
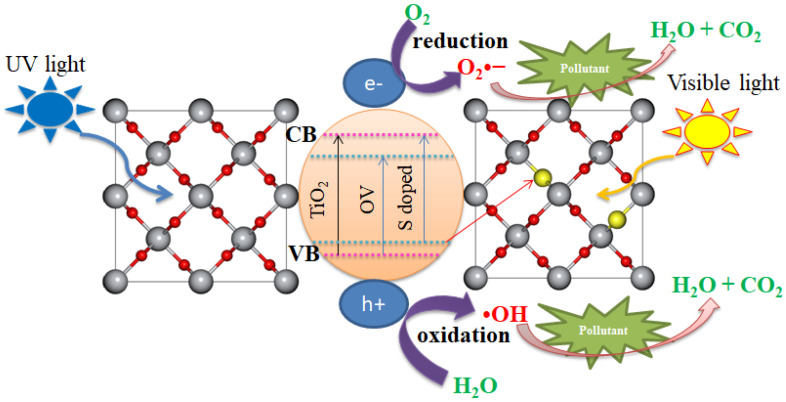
Photocatalytic mechanisms of S-doped rutile TiO_2_.

The photocatalytic mechanism of S doped rutile is shown in Figure 12. When S-doped rutile TiO_2_ is exposed to light, electrons in the valence band are excited to the conduction band, creating electron–hole pairs, as given in Equation (23).

(23) $$Sdoped\;TiO_{2}+h\nu \to e^{-}+h^{+}$$

The photogenerated charge carriers are essential for the photocatalytic degradation of pollutants. The S induced energy states within the bandgap reduce recombination rates of electron–hole pairs, thereby enhancing the overall photocatalytic efficiency. The photo-generated electrons in the conduction band can be captured by oxygen molecules, forming superoxide anion radicals (O_2_•^−^), while the holes in the valence band can oxidize water or hydroxide ions to produce hydroxyl radicals (•OH), as given in Equations (24) and (25), respectively.

(24) $$O_{2}+e^{-}\to O_{2}{•}^{-}$$

(25) $$H_{2}O+h^{+}\to •OH+H^{+}$$

Both of these reactive oxygen species (ROS) play a critical role in the degradation of organic pollutants. The presence of S doping can help stabilize the reactive oxygen species, enhancing the efficiency of pollutant degradation [54]. The ROS attack organic pollutants like dyes, pharmaceuticals, and pesticides in the wastewater, breaking them down into smaller, less harmful molecules, such as H_2_O and CO_2_.

## 4. Conclusions

In this study, we investigated the effects of sulfur (S) doping and oxygen vacancy (OV) creation on the physical properties of rutile TiO_2_, with a particular focus on their implications for photocatalysis applications. Through density functional theory plus Hubbard parameter (DFT + U) calculations, we explored how S doping and OV formation influence the structural, electronic, mechanical, and optical properties of rutile TiO_2_. The structural analysis reveals that the unit cell of TiO_2_ expands upon S doping, primarily due to the difference in atomic radii between sulfur and oxygen atoms. This expansion is observed along both the a and c axes, and the bond lengths increase after S incorporation into the TiO_2_ lattice. In contrast, the introduction of oxygen vacancies causes shrinkage in the unit cell as a result of the missing oxygen atoms. The analysis of electronic properties demonstrates that both S doping and OV creation effectively reduce the bandgap of TiO_2_, shifting it from the UV region to the visible light spectrum. This bandgap reduction significantly enhances the photocatalytic activity of TiO_2_ under visible light irradiation, making it more efficient for pollutant degradation and other photocatalytic applications. Mechanical property analysis highlights a trade-off: while S doping strengthens the material’s rigidity, oxygen vacancies decrease its mechanical strength compared to S doping. Additionally, both S doping and OV formation result in a red shift in the absorption spectrum, which further confirms their potential to improve the light absorption capabilities of TiO_2_, particularly in visible-light-driven photocatalytic processes. These findings provide valuable insights into designing photocatalysts with enhanced photocatalytic performance and stability. The ability to tailor TiO_2_’s structural, electronic, and mechanical properties through doping and vacancy engineering holds great promise for advancing solar energy conversion and other optoelectronic technologies. Overall, this study contributes to the development of more efficient, durable, and practical photocatalysts for environmental and energy-related applications. Finally, based on our current DFT findings, we recommend conducting experimental work with the same concentrations of S dopants, as it is crucial for practical photocatalysis applications.

## Figures and Tables

**Table 3:** Formation energy of S-doped TiO_2_ and TiO_2_ with oxygen vacancy.

Systems	S Doping at Ti Sites	S Doping at O Sites	6.25% Doped TiO_2_	12.5% Doped TiO_2_	18.75 Doped TiO_2_	OV
Formation energy (eV)	2.73	−3.07	−4.59	−3.07	−1.54	−1.68

**Table 4:** Calculated elastic constants (*C_ij_*), bulk modulus (*B_H_*), Young’s modulus (Y), shear modulus (*S_H_*) in GPa, and Poisson’s ratio (*n*) of undoped TiO_2_, S-doped, and oxygen vacancy.

Materials	*C*_11_	*C*_12_	*C*_13_	*C*_33_	*C*_44_	*C*_66_	*B_H_*	Y	*S_H_*	*n*
Undoped TiO_2_	253.2	128.7	129.2	435.1	116.8	197.1	185.1	285.4	114.8	0.24
Other DFT for TiO_2_ [43]	271	143	144	465	124	188	208	303.6	111	0.25
Exp. for TiO_2_ [34]	268	175	147	484	124	190	212	--	113	--
6.25% doped	266.4	243.3	159.9	303.1	365.1	271.4	199.3	298.4	119.3	0.27
12.5% doped	273.2	245.6	167.4	324.1	369.2	283.7	301.9	319.5	120.6	0.32
18.75% doped	291.3	253.2	174.6	341.7	374.4	286.3	305.2	314.3	118.3	0.53
O-Vacancy	119.0	111.4	117.9	176.0	109.2	115.3	157.1	254.1	103.3	0.46

**Table 5:** The obtained optical properties at static (0 eV) and maximum values.

Materials	$\varepsilon_{r}\left(0\right)$	$n\left(0\right)$	R(0)	$L_{max}\left(\omega \right)$	$\alpha_{max}\left(\omega \right)$ $\left(10^{27}/cm\right)$	$\sigma_{max}\left(\omega \right)$ ${\left(10^{35}\times Ω.cm\right)}^{-1}$
undoped TiO_2_	8.88	2.97	0.25	1.9	1.23	5.22
6.25% doped	22.77	4.77	0.42	2.1	1.24	6.36
12.5% doped	15.34	3.91	0.35	2.8	1.57	9.08
18.75% doped	4.51	8.89	0.79	3.2	1.70	8.39
Oxygen vacancy	4.41	2.09	0.12	1.3	0.87	2.93

## Data Availability

The data that support the findings of this study are available from corresponding author upon reasonable request.
